# Inkjet Printing Functionalization of SOFC LSCF Cathodes

**DOI:** 10.3390/nano9040654

**Published:** 2019-04-24

**Authors:** Eleonora Venezia, Massimo Viviani, Sabrina Presto, Vasant Kumar, Rumen I. Tomov

**Affiliations:** 1 Department of Chemical, Civil and Environmental Engineering, Università degli studi di Genova, 16146 Genova, Italy; 2 CNR-ICMATE, c/o DICCA-UNIGE, 16145 Genova, Italy; massimo.viviani@ge.icmate.cnr.it (M.V.); sabrina.presto@ge.icmate.cnr.it (S.P.); 3 Department of Materials Science and Metallurgy, University of Cambridge, Cambridge CB2 1TN, UK; rvk10@cam.ac.uk (V.K.); rit21@cam.ac.uk (R.I.T.)

**Keywords:** inkjet printing, infiltration, nanoparticles, solid oxide fuel cells

## Abstract

An important segment of the future renewable energy economy is the implementation of novel energy generation systems. Such electrochemical systems are solid oxide fuel cells, which have the advantage of direct conversion of the chemical energy stored in the fuel to electrical energy with high efficiency. Improving the performance and lowering the cost of solid oxide fuel cells (SOFCs) are strongly dependent on finding commercially viable methods for nano-functionalization of their electrodes via infiltration. Inkjet printing technology was proven to be a feasible method providing scalability and high-resolution ink delivery. La_x_Sr_1−x_Co_y_Fe_1−y_O_3−δ_ cathodes were modified using inkjet printing for infiltration with two different materials: Gd-doped ceria (CGO) commonly used as ion-conductor and La_0.6_Sr_0.4_CoO_3–δ_ (LCO) commonly used as a mixed ionic electronic conductor. As-modified surface structures promoted the extension of the three-phase boundary (TPB) and enhanced the mechanisms of the oxygen reduction reaction. Electrochemical impedance measurements revealed significantly lowered polarization resistances (between 2.7 and 3.7 times) and maximum power output enhancement of 24% for CGO infiltrated electrodes and 40% for LCO infiltrated electrodes.

## 1. Introduction

The commercialization of solid oxide fuel cells (SOFCs) is reported to be deterred by their high production costs, as well as operational losses, sourcing largely from the high operational temperature. Temperatures above 800 °C assume a restrictive choice of applicable interconnects and sealing solutions. Such temperatures can reduce the longevity of the SOFC stacks causing deterioration of various stack components [1]. Significant efforts are directed towards the reduction of operational temperatures below 800 °C. This would allow for the utilization of cheaper steel interconnects and cell supports, as well as application of commercially available sealing techniques. The expected outcome has a substantial cost reduction and improved reliability. It would lower the stack degradation resulting from possible inter-diffusion reactions and compositional alterations. Critically, it would also enable faster thermal cycling and start up [2,3].

The rate-limiting chemical reactions occurring at SOFC electrodes are thermally activated which leads to an exponential decrease in the reaction rates and overall reduction of SOFCs electrochemical performance. The reduction of cathode polarization losses is found to be of paramount importance, as the activation energy of oxygen reduction reaction (ORR) [4,5] is generally higher. Nano-decoration (infiltration) with reaction promoters is an effective route for the enhancement of cathodes electro-catalytic activity at lower temperatures, as a result of an extended three-phase boundary (TPB) density and accelerated ORR adsorption/dissociation mechanism [6].

The goal of this work was to demonstrate the applicability of the inkjet printing technology (IJP) for a controllable and scalable infiltration nano engineering of the SOFC cathodes. An ionic conductor, Ce_0.9_Gd_0.1_O_2−x_, and a mixed ionic electronic conductor, La_0.6_Sr_0.4_CoO_3−δ_, were infiltrated to create a nano particle coverage of La_0.6_Sr_0.4_Co_0.2_Fe_0.8_O_3−δ_ (LSCF) porous skeleton. Parallel infiltration procedures were studied in a comparative study.

## 2. Infiltration Strategies

**Figure 1:**
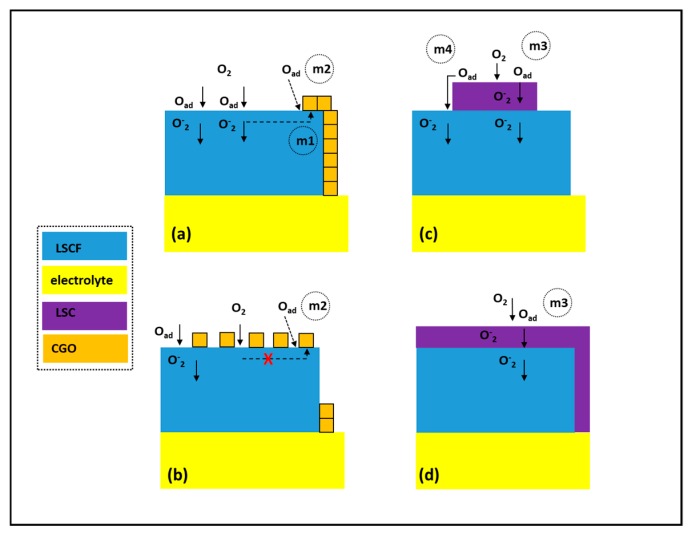
Possible mechanisms contributing to the O_2_ redox reaction by infiltration of (**a**,**b**) ion conductive phase (e.g., Gd-doped ceria (CGO) (m1—extension of three-phase boundary (TPB); m2—adsorption/dissociation at TPB)) and (**c**,**d**) mixed ionic electronic conductor (MIEC) phase (e.g., La_0.6_Sr_0.4_CoO_3−δ_ (LSC) (m3—adsorption/dissociation; m4—spillover migration)).

The commonly used infiltration strategies could be classified by the type of infiltrated promoter introducing different functionality (e.g., by the type of the conductivity—ionic or mixed ionic electronic) or by the scaffold being infiltrated—either single phase (e.g., LSCF) or composite material (e.g., LSCF/doped ceria). Infiltration of a monophasic type of scaffold with an ion-conducting phase generally requires higher loading levels to provide percolation of the infiltrated nanoparticles and formation of an extended TPB (Figure 1a(m1)). Low infiltration levels usually lead to lack of interconnectivity between the infiltrated nanoparticles and hence no extension of the TPB. The boundary between the infiltrated nanoparticles and the mixed ionic electronic conductor MIEC scaffold could, in some cases, provide sites with enhanced surface exchange coefficient introducing an accelerated adsorption/dissociation mechanism—m2. Adversely, an extensive masking of the MIEC catalytic surface with an ion-conducting phase possessing low catalytic activity can lead to an overall drop of cathode performance (Figure 1b). In the case of MIEC promoter, any loading levels of infiltration can lead to a substantial change in the electrochemical performance via promotion of additional mechanisms of adsorption/dissociation activity (Figure 1c (m3,m4)). A complete coverage of the MIEC scaffold with more a catalytically active MIEC promoter should not have a negative effect, except in cases of very high loading levels where appearance of concentration polarization losses can be expected (Figure 1d).

The low calcination temperature (<800 °C) of the infiltrated precursors permits flexibility in the choice of infiltrate materials, which could be otherwise incompatible with conventional ceramic processing due to either high reactivity and/or mismatch of the thermal expansion coefficients. Numerous variations of scaffold/infiltrate architectures have been described in several review articles [7,8,9]. The choice is generally governed by the pursued goals—enhancement of the electro-catalytical performance and/or improvement of electrode durability/compatibility.

Different types of surfactants are often used to control the scaffold wetting aiming at better dispersion uniformity and size control of the infiltrated nano-particles (Triton-X100 and X-45 [10,11], 2-butoxyethanol [12], Pluoronic P123 [13]). Although, generally the surfactants were found to play a positive role in solution based synthesis, but some contradicting reports on their use were published. According to Lou et al. [14] surfactants like Triton X-100 and X-45 do not improve the quality of the infiltrated promoters like Sm_0.5_Sr_0.5_CoO_3−δ_ (SSC) or LSCF into the cathode scaffold. Klemensø et al. [15] found that the presence of surfactants had an insignificant influence on the electrochemical performance of the cell as related to the changes in the cathode surface alteration. It was pointed out that rapid calcinations could be beneficial in preventing the coarsening of the nano decoration.

An alternative way of controlling the wetting properties is synthesizing inks based on organic solvents. It was previously observed that EtOH based ink was shown to achieve a high degree of wetting on both metal and oxide surfaces without utilization of additional surfactants [16].

Performing comparative analyses on published data on the effects of SOFC cathodes infiltration is difficult due to the lack of convention on a figure of merit. The variation of experimental parameters is very wide and includes several different groups of parameters—structural parameters (type and porosity of the electrode scaffolds), methodological parameters (loading levels, number of infiltration, use of vacuum, calcination procedure), technological parameters (ink rheology, use of surfactants, testing configuration), etc. All of the above can contribute to the final measurable result. In order to evaluate the published data, some relative promotion factors have been formulated. Several promotional factors (*F*) were introduced reflecting on the improvement of the infiltrated cathode over a reference non-infiltrated (blank) one:

*F_ASR_* = *ASR*_blank_/*ASR*_inf_ OR *F_p_* = *P_max-inf_*/*P_max-blank_* (1)

where area specific resistance (*ASR*) or maximum output power (*P*) are used as measurable variables.

## 3. Infiltration of Ion Conductive Phase

Due to its high ionic conductivity and compatibility with cobaltite-based cathode compounds, doped ceria is commonly used as infiltrated promoter for SOFC cathodes [17]. LSCF cathodes have been commonly used at intermediate SOFC operational temperatures due to a number of physicochemical advantageous parameters, like high surface exchange and bulk diffusion coefficients, as well as ionic and electronic conductivity [18,19]. Adversely, cobaltite-based cathodes were shown to be prone to significant long-term degradation of ~0.05% per hour [20]. One generally recognized degradation mechanism as the preferential segregation of *Sr* onto the LSCF surface, which obscures the sites available for the ORR leading to lower surface activity. An obvious way to alleviate such deterioration is to reduce the operational temperature and hence prolong the lifetime of the cell. This would require finding a compensational mechanism for the thermally reduced catalytic activity of the electrode. A simple solution for the problem is extending the density of the TPB with an infiltrated ion conductive nano decoration, resulting in a reduction of the polarization losses as shown in Figure 1a. A significant number of experimental studies on doped ceria infiltration into cobaltite-based cathodes have been published over the last decade. A summary of some typical *F_ASR_* values measured in the region of 600–800 °C is presented in Table 1. In general, the reported promotion factors tended to be higher at lower testing temperatures. Such an increase in the catalytic activity of LSCF cathodes was reported also for low loading levels where infiltrated doped ceria nano particles did not form percolation nano decoration (Figure 1b). An explanation of such an effect was given by Xia at al. [21,22] who reported that coverage of LSCF surface with discrete Sm-doped ceria nano particles can increase the surface exchange rates by a factor of 10. The surface exchange coefficients of LSCF/Sm_0.2_Ce_0.8_O_2-x_ (SDC)/gas TPB were found to be higher than that of the LSCF/gas boundary due to the contribution of additional free vacancies contributed by the infiltrated doped ceria.

## 4. Infiltration of MIEC Phase

Different cobaltite compounds such as Sm_0.5_Sr_0.5_CoO_3−δ_ (SSC), La_0.6_Sr_0.4_CoO_3−δ_ (LSC), SrCo_0.8_Fe_0.2_O_3−δ_ (SCF), and Ba_0.5_Sr_0.5_Co_0.8_Fe_0.2_O_3−d_ (BSCF) [27] possess superior electrochemical activity in comparison to LSCF—higher bulk oxygen ion diffusion coefficient and/or higher surface oxygen exchange rate. Adversely, they have thermal expansion coefficients, which are not compatible with yttrium stabilized zirconia YSZ and Gd-doped CeO_2_ electrolytes [28]. This fact, along with their high reactivity with zirconia-based electrolytes, renders those MIEC materials technologically difficult to implement. The use of composite cathodes (e.g., LSCF/ Gd-doped ceria (CGO)) has beneficial effects on both performance and stability [29] although some degradation is still present after long operation. However, the formation of nano-structured decorations via infiltration and low temperature calcinations is an effective approach ensuring the contribution of highly active catalyst material without compromising the cathode stability.

Ai et al. [30] fabricated nanostructured cathodes by incorporating BSCF impregnation into a conventional La_1−*x*_Sr*_x_*MnO_3_ (LSM) electrode. A loading level of ~1.8 mg·cm^−2^ led to the reduction of polarization resistance (Rp) values at 800 °C by a factor of *F_ASR_* = 12. The incorporation of the nanosized BSCF particles not only increased the electrocatalytic activity but also was reported to enhance the stability of the LSM cathode. Xu et al. [31] performed one-step infiltration of LSC and SCF into a commercial La(Ca,Ce)Mn(Ni,Cr)O_3_ cathode and reported very high promotional factors for LSC infiltrate (*F_ASR_* = 17−28) and SCF infiltrate (*F_ASR_* = 28−49). Lou et al. [14] used one-step infiltration to deposit 50–100 nm thin film of SSC on LSCF cathode skeleton. As the infiltrated SSC is catalytically more active than LSCF, the infiltration led to a significantly reduced Rp (*F_ASR_* = 15.15 at 550 °C). The infiltrated nano-coatings were shown to maintain the stability of LSCF cathodes.

## 5. Methodology

Inkjet printing (IJP) of functional materials has become a widely used technique in scientific research in recent years [32,33], although its commercialization began much earlier—in the second half of the 20th century when the technology was implemented for computer graphics output. Logically, the graphical reproduction remains its main conventional application. In recent decades however, the success of nanotechnology led to various attempts to utilize IJP for the nano-functionalization of various materials. This was driven by the obvious advantages of IJP related to the synthesis of nanomaterials: controllable delivery and accurate positional placement; suitability for dispensing a wide range of materials; minimized wastage of expensive precursors; non-contact nature providing an opportunity to work on a variety of substrates (including fragile, flexible, patterned, or reactive ones) and with nanomaterials sensitive to mechanical pressure; avoidance of lithographic techniques and hence a significant reduction of production and capital costs.

A considerable amount of research and commercialization efforts have been concentrated on energy related devices: thin-film photovoltaics, supercapacitors, batteries, SOFC and polymer electrolyte membrane fuel cells (PEMFC), etc. A variety of IJP technologies based on different physical principles have been applied in functional materials printing—piezoelectric, electrostatic, thermal, solenoid valve, etc. [34]—all having distinct advantages, fundamental limitations, and principles employed to eject ink drops. Nevertheless, a number of stages involved in IJP are commonly defined: (i) generation and transfer of drops to the substrate surface; (ii) interaction of the drops with the substrate, and (iii) overlapping of the drops and their solidification/drying. Each of those stages has its own constraints and parameter optimization windows in which the functionalization of nanomaterials is most effective.

A critical issue in the infiltration nano-engineering of SOFC electrodes is the extra cost added to production by the additional steps. The bulk of laboratory-based experimentation is based on simple infiltration methods like immersion in or pipetting of the ink. Depending on the desired loading levels, the infiltration could be performed in several steps with/without assistance of a vacuum. Such treatments are not scalable and offer little control over the uniformity of the ink distribution and the wastage of ink. In an effort to make the procedure more feasible, Lee et al. [35] utilized consecutive foam roller applications to infiltrate the ink into SOFC anode functional coatings. A different approach was adopted by Kiebach et al. [36]. A simple “flashing” of the stack manifold with aqueous inks was used to produce significant performance improvement of the SOFC stack. Dowd et al. used aqueous LSC infiltrate solution to infiltrate LSCF/SDC cathodes by spraying the cathode’s surface with an atomized ultrasonic nozzle [37]. All of those approaches have potential for an industrial scale-up although at the expense of irreversible ink losses and unpredictable non-uniformity of the infiltrate distribution. Mitchell-Williams et al. [38] and Tomov et al. [26] recently proved the feasibility of IJP as a non-disruptive technique for ink infiltration into porous SOFC anodes and cathodes. IJP offers the opportunity for a low-cost industrially scalable infiltration and is capable of precise delivery of pico– to nano-liter drops with micron-scale lateral resolution. The latter opens feasible perspectives for use of rare earth or noble metals as ORR reaction promoters.

Inkjet printing technologies can be divided into two main categories: drop on demand (DOD) and continuous inkjet (CIJ) printing. CIJ is mostly used for low-quality coding and marking applications. DOD allows better control over droplet positioning and so it is the preferred technique for high-resolution graphics and materials inkjet printing. The three most common technologies for DOD drop generation are thermal, piezoelectric, and electromagnetic (valve jet). Thermal (or bubble-jet) printing imposes severe restrictions on ink formulation and is thus generally avoided for materials deposition. The electromagnetic and piezoelectric approaches are therefore strongly favored. Piezoelectric droplet formation is the main DOD technique used in most industrial inkjet print heads. The droplet volume depends on the diameter of the orifice and is usually in the 1–100 pL range. Piezoelectric print heads usually contain piezoelectric actuators, which reduce the internal volume of the chamber to dispense a droplet. This introduces restrictions on the choice of ink carrier and makes it particularly difficult to produce reliable print head for aqueous inks (e.g., to separate the electrodes and actuators from the ink). Nevertheless, print heads with suitability for aqueous ink types are available on the market. Piezoelectric tube actuators can also be bonded to the wall of an ink-carrying capillary to induce an acoustic or pressure wave to provoke droplet formation. This has the advantage of eliminating contact between ink and actuator, but makes it difficult to produce high nozzle density print heads. This technique is therefore used in single-nozzle print heads, micro-dispensers and micro-pipettes.

For electromagnetic solenoid micro-valve (valve-jet) printing, a chamber with a nozzle at one end is occupied by a plunger assembly, with a component to seal against the nozzle at one end and a magnetic core at the other. Pressurized ink is introduced, and a solenoid is used to displace the plunger (opening the valve) when jetting is required. The drop size is controlled by the orifice diameter, the time for which the valve is kept open, and the chamber pressure. Valve-jet technology is compatible with a wider range of inks (both solutions and suspensions). Additional advantages of the electromagnetic printing heads are their robustness, easier maintenance, and lower cost. A summary of the basic characteristics of the piezoelectric and the electromagnetic printing technologies are presented in Table 2.

**Figure 2:**
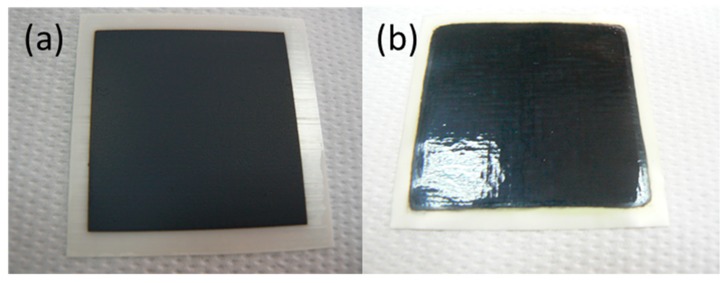
LSCF cathodes inkjet printed (**a**) and infiltrated with Gd-doped ceria (CGO) (**b**) on a large scale (50 × 50 mm) 150 µm thick 3YSZ electrolyte substrate.

The choice between piezoelectric and electromagnetic DOD printing for a particular task depends mainly on the required resolution and the characteristic of the ink. Valve jet technology produces significantly larger drops (10–50 nL) which are perfectly suitable for the majority of materials research jobs not requiring high resolution patterns. Considering the specific requirements of the SOFC electrodes infiltration procedure and the advantages/disadvantages of the main printing, technologies a choice has been made to study the feasibility of the electromagnetic print heads for the infiltration nano-engineering of SOFC electrodes. The size of the cells experimented with in this study (11 to 20 mm diameter) necessitated the use of a single micro-valve nozzle (Domino Macrojet). Generally, there are no practical restrictions on the size of the printable area using multi-nozzle print heads. Figure 2 provides an example of utilization of a 16-nozzle Domino Macrojet print head for deposition of LSCF cathode on 100 µm thick 3YSZ electrolyte substrate (from suspension ink) (Figure 2a) and the consequent infiltration of a similar cathode with CGO ink.

## 6. Experimental

Ionic conductor precursor ink (CGO) or MIEC precursor ink (LSC) ink were jetted in nanoliter volume drops with velocity of several m/s before impact with the porous surface of the electrode. The resulting momentum forced controlled amount of inks to permeate the electrode. Three printing passes were performed over the cathode of commercial LSCF (30 µm) |3YSZ(100 µm)|NiO-CGO (30 µm)—button cells (CEREL, Boguchwała, Poland). After the infiltration, a calcination at 700 °C in air was applied.

A durability study was performed on LSCF/CGO/LSCF symmetrical cells infiltrated in an identical manner. The cells were based on CGO dense electrolyte pellets with diameter of ~11 mm and thickness of ~0.5 mm. The procedure of the cathode electrodes preparation using LSCF suspension ink is described elsewhere [26].

For CGO ink a stoichiometric quantity of cerium nitrate hexahydrate (99.999%, Alfa Aesar, Haverhill, MA, USA) and gadolinium nitrate hexahydrate (99.9%, Alfa Aesar, Haverhill, MA, USA) were added to absolute ethanol. Urea (>99.5%, Fisher Scientific, Hampton, NH, USA) was added as a complexing agent (1:1.5 molar ratio metals:urea). The powders were dissolved with stirring and heating at 40 °C. The solution was made up to 0.75 M total metal ion concentration ink with absolute ethanol and filtered before storage. The LSC ink was prepared in the same way and with the same concentration. A programmable viscometer (LVDV-II+, Brookfield, Toronto, ON, Canada) with rotation speeds spanning from 20 to 160 rpm, was used for the viscosity measurements.

The electrochemical characterization of the samples was performed with electrochemical impedance spectroscopy (EIS). Pt mesh current collectors were placed in contact with the working and counter electrodes in a two-electrode configuration connected to a potentiostat frequency response analyzer (IVIUMSTAT, Ivium Techologies, Eindhoven, The Netherlands). Impedance measurements were performed in H_2_ and O_2_ atmosphere at open circuit voltage (OCV) in 0.1 Hz–100 kHz frequency range. I–V characteristics of the cells were tested in a vertical ProboStat test rig exposing the cathode side of the cell to a mix of oxygen and nitrogen (20 and 15 mL/min, respectively). Humidified hydrogen (25 mL/min) was flown on the anode side.

In order to illuminate the cathode surface evolution, fractured cells’ cross-sections were studied with SEM (15 kV acceleration voltage). For the structural characterization X-ray diffraction (XRD) was applied with Bragg–Brentano geometry using a D8 Advance Bruker diffractometer.

## 7. Results and Discussions

### 7.1. Inkjet Printing Infiltration

**Figure 3:**
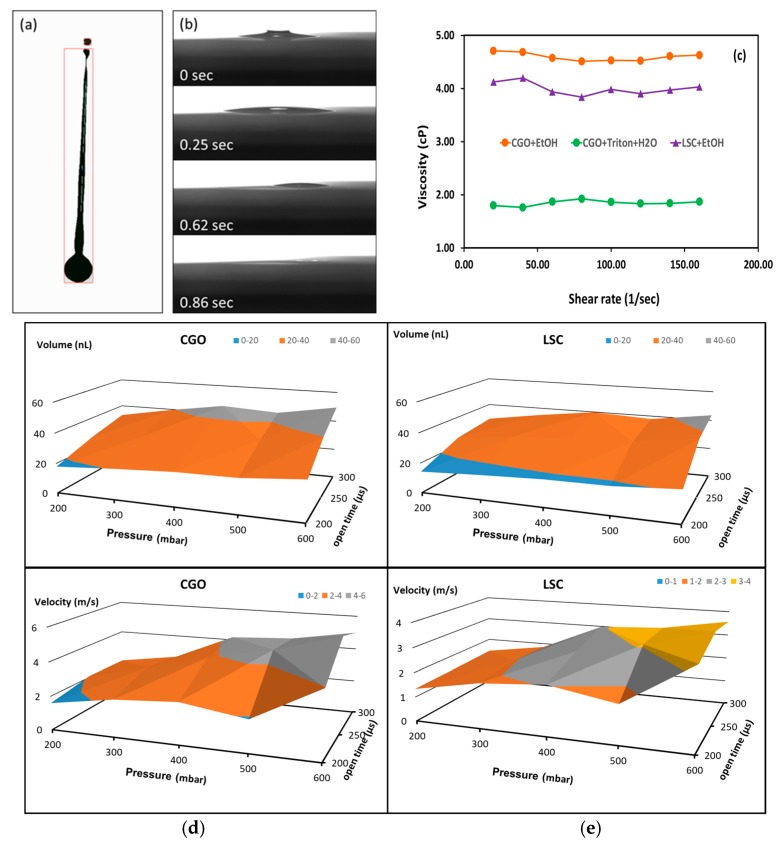
(**a**) Jetting behavior of CGO ink; (**b**) permeation dynamics of CGO ink drop infiltrated in LSCF scaffold; (**c**) viscosity measurements of both infiltrate inks (water-Triton X-100 based CGO ink viscosity data is presented for comparison) and volume/drop velocity dependences on printing parameters for both (**d**) CGO and (**e**) LSC inks.

Micro-valve nozzle (Domino Macrojet) IJP system based on a print head scanning in the X–Y plane of the cell was employed for infiltration of the LSCF cathodes. The print head jetted ink drops within a tunable volume range of 10–80 nL. A drop visualization system [39] was employed to determine the optimum jetting parameters for a production of drops with necessary volumes and velocities to avoid the formation of satellite drops. An image illustrating a single CGO ink drop formation is shown in Figure 3a. At the beginning of the jetting event an elongated drop is detached from the nozzle plate accompanied by satellite drops. An adjustment of the nozzle opening time and pressure can lead to merging the latter with the main drop before reaching the surface of the substrate. More detailed information on the optimization procedure, as well as the interaction of the drops with the porous electrode, is reported elsewhere [40]. Figure 3b demonstrates the dynamic of a single drop penetration into the porous LSCF scaffold. The optimal distance between the drops landings on the scaffold was empirically established to be 1 mm. The full penetration of the ink was achieved for 0.86 s (Figure 3b). As the time required to overprint the areas of the cathodes was between 15 and 25 s (depending on the type of the cell), this assured that the ink had permeated into the scaffold completely before the next printing pass over the same area was executed. Thus, an overloading of the scaffold with ink was avoided ensuring a uniform lateral distribution of the ink. Three printing passes were made without overloading. Any further increase of the infiltrate mass load required heat treatment in order to evacuate the solvent from the pores of the cathode. As one of the goals of the study was to minimize the cost of the procedure, the infiltration was limited to three passes and referred hereafter as a “single-step” infiltration.

Figure 3d,e illustrate the range of variations of the drop volumes and velocities over the studied printing parameters range of stable jetting (without formation of satellite drops or splashing). The choices of optimized key printing parameters were made in such a way as to produce similar drop volumes and velocities leading to similar molar loading of both materials as shown in Table 3.

**Figure 4:**
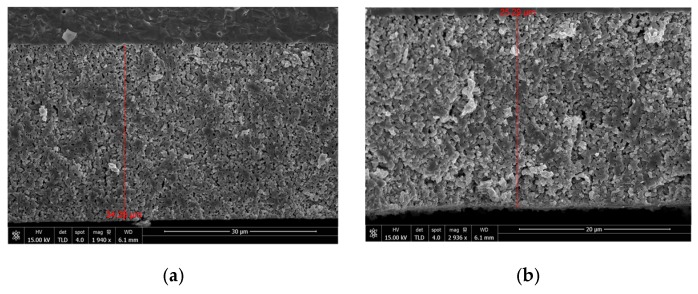
CGO ink distribution within LSCF porous scaffold for drop velocity of (**a**) 2.3 m·s^−1^and (**b**) 3.7 m·s^−1^.

In order to check the influence of drop velocity on the depth of ink permeation into the porous scaffold, cross-sectional SEM images were taken of two LSCF cathodes inkjet infiltrated with CGO at two different drop velocities (2.3 and 3.7 m/s) with the same amount of ink (~15 μL/electrode). As shown in Figure 4 similar uniform distribution was registered in both cases suggesting that, for the present porosity/particle size range of the scaffold, the process of ink penetration is mostly driven by the ink rheology.

### 7.2. Characterization

**Figure 5:**
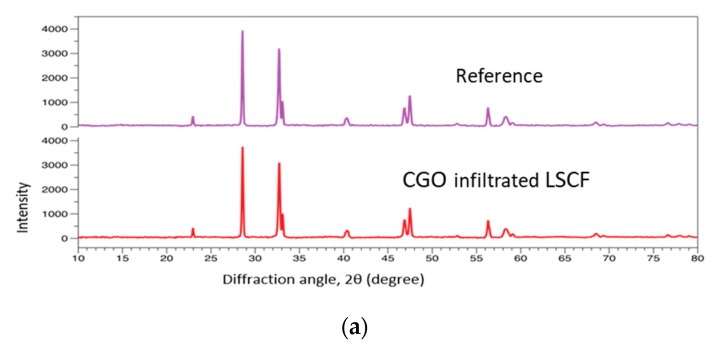

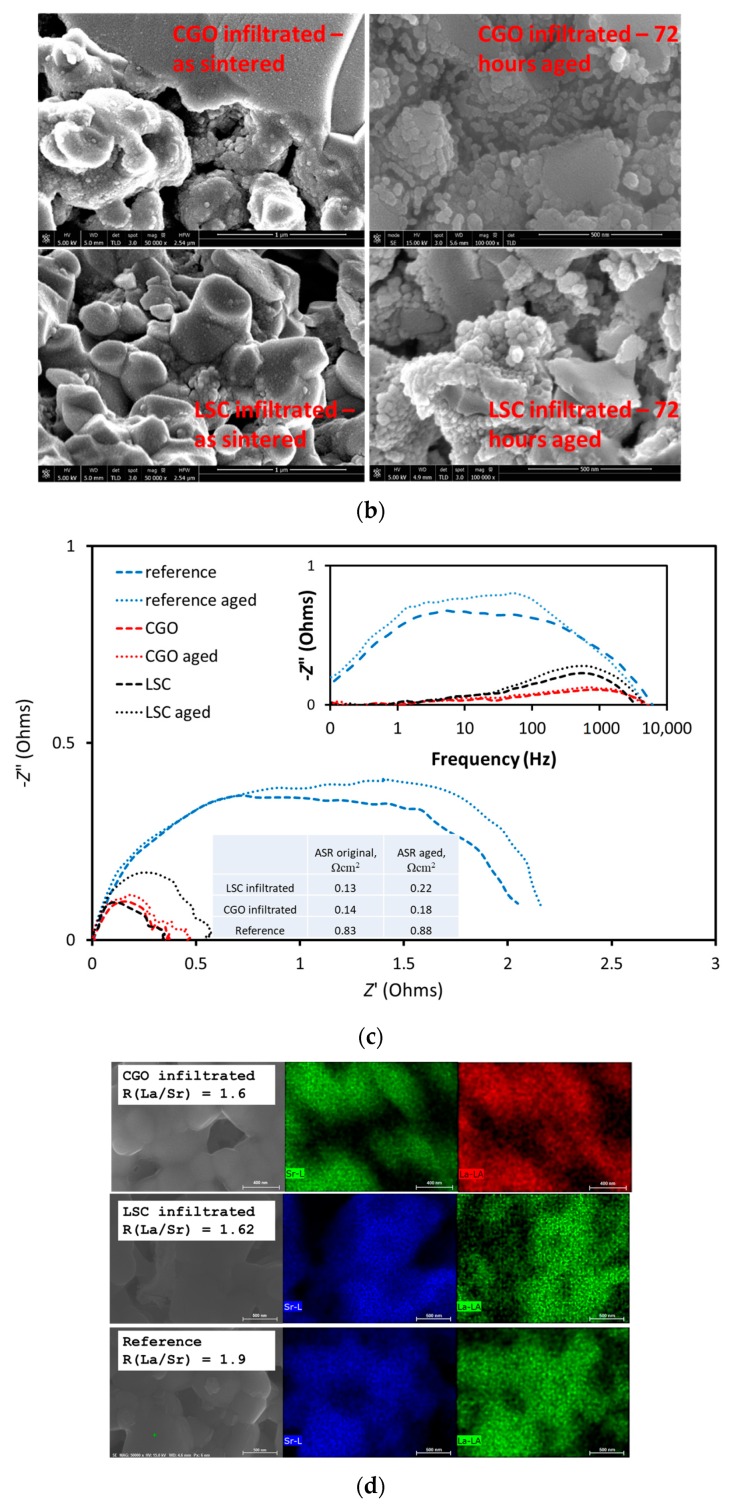
(**a**) XRD of the reference and the calcined CGO-infiltrated cell; (**b**) microstructural comparison of infiltrated cells before and after aging (**c**) EIS aging testing data at 700 °C in air; (**d**) EDX mapping of La and Sr after 72 h of aging.

The symmetrical LSCF/CGO/LSCF were infiltrated with CGO and LSC inks in an identical procedure. XRD study of the reference and the CGO infiltrated cathode did not find any detectable structural differences between these after calcination at 700 °C (see Figure 5a). This observation was in agreement with previously reported data that the interaction between LSCF and CGO at these temperatures is negligible due to the low solubility of Ce and Gd in LSCF [41]. SEM cross-sectional images of symmetrical LSCF/CGO/LSCF cells infiltrated with CGO and LSC inks (as-sintered and after 72 h of testing (aging)) are presented in Figure 5b. The total nanoparticle loading was ~10 wt %. The images of the as-sintered cell reveal uniform coverage of the scaffold with fine nanoparticles. After an Electrochemical Impedance Spectroscopy EIS testing for 72 h the CGO particles tended to form characteristic “chains” of larger nanoparticles approximately 30 nm in size, while the LSC infiltrate was clearly aggregated in large “rug–type” formations of nanoparticles approximately 50–100 nm in size. EIS tests were done at 700 °C in air before and after aging for 72 h. The relevant Nyquist and Bode plots for the reference and the infiltrated cathodes (see Figure 5c) reveal a significant deterioration of the catalytic properties of the reference electrode due to the increase in low frequency losses. The effect was assigned to Sr surface segregation. At the same time, the change in the polarization resistances of infiltrated cells was much smaller in comparison, with a more substantial increase in high frequency losses for the LSC infiltrated cell. The estimated *ASR* values for the reference electrode and the infiltrated electrodes before and after aging are summarized in the inset of Figure 5c. While the *ASR* for the LSC infiltrated cathode showed an increase of ~65%, the CGO electrode was significantly more stable (~30% increase in *ASR*). Energy-dispersive X-ray spectroscopy (EDX) point mapping of La and Sr of the scaffold produced after 72 h of aging is presented in Figure 5d. It confirmed a significant Sr depletion of the LSCF grain volume (La/Sr) at % ratio of ~1.9 in comparison with (La/Sr) at % ratio of ~1.6 for the infiltrated cells, which was close to the original LSCF powder value.

A previously published experimental study on the infiltration of CGO and Co_x_O_y_ inks into composite LSCF/CGO cathodes revealed a substantial Sr segregation suppression during the aging of infiltrated electrodes. While a high concentration of strontium in the form of SrCO_3_ was observed on the surface of the reference electrode by high-resolution X-ray photoelectron spectroscopy (XPS), the infiltrated surfaces were shown to have approximately an order of magnitude less surface associated SrCO_3_ species. This stability effect was ascribed to the surface modification introduced by the nano infiltration. Although the mechanism of Sr segregation is not clearly understood, the high mobility of Sr [42] and the surface charge effects [43] are commonly considered as underlying factors. In a first principles study of thermodynamic driving forces Ding et al. [20] suggested reducing surface charges via depositing additional surface coatings for suppression of Sr surface segregation. A number of experimental studies confirmed the validity of this hypothesis [44,45].

**Figure 6:**
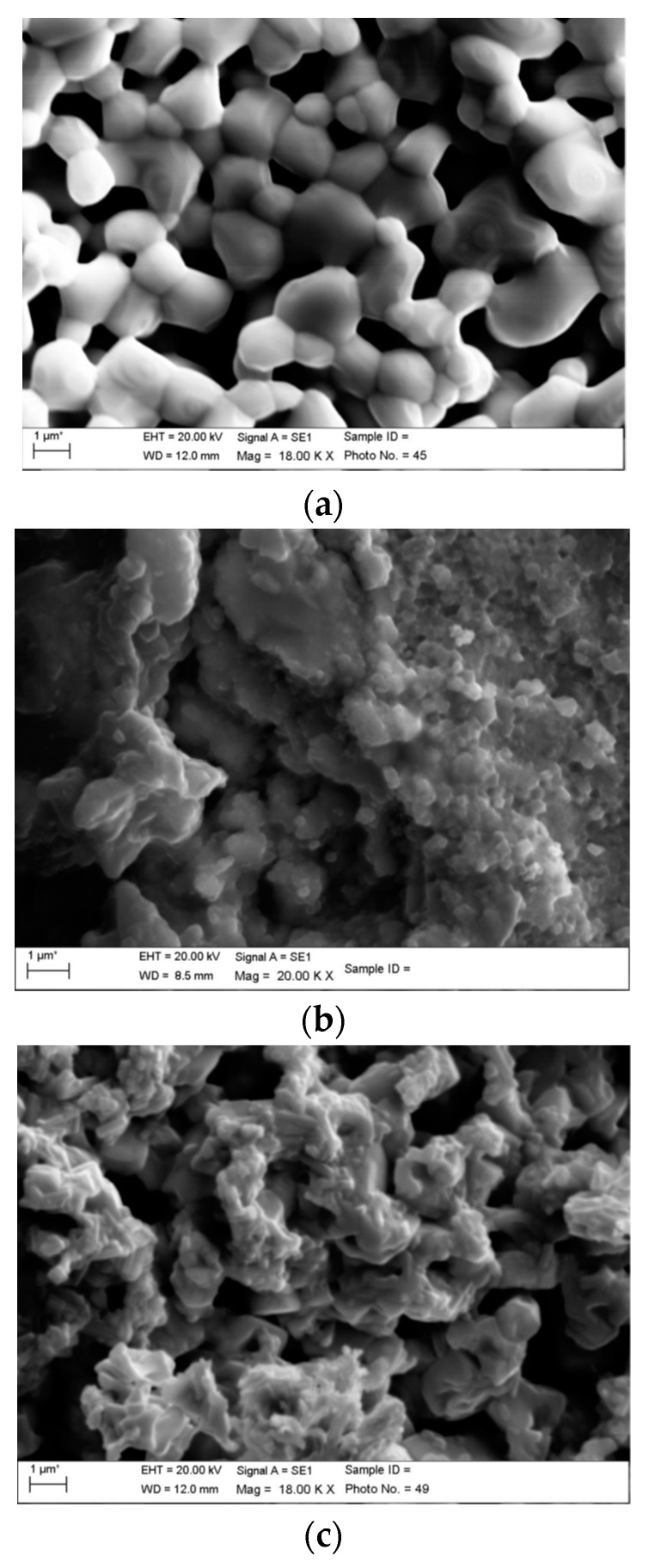
SEM cross-section images of the reference and the infiltrated samples after EIS characterization. (**a**) LSCF; (**b**) CGO-LSCF; (**c**) LSC-SrCo_0.8_Fe_0.2_O_3−δ_ (SCF).

Infiltration of full button electrolyte cells was performed in an identical manner. The morphology of the reference, CGO infiltrated cathode and LSC infiltrated cathodes are shown in Figure 6a–c, respectively. CGO-infiltrated cathode showed percolating decoration of discrete nanoparticles throughout the whole electrodes volume. Nanoparticle sizes between 10–50 nm were measured. In contrast, LSC infiltrate was present as a continuous coating of LSC on the surface of the electrode. The observed nanoscale decorations could lead to a TPB extension as well as to enhancement of the catalytic activity by introducing supplementary mechanisms for the ORR.

**Figure 7:**
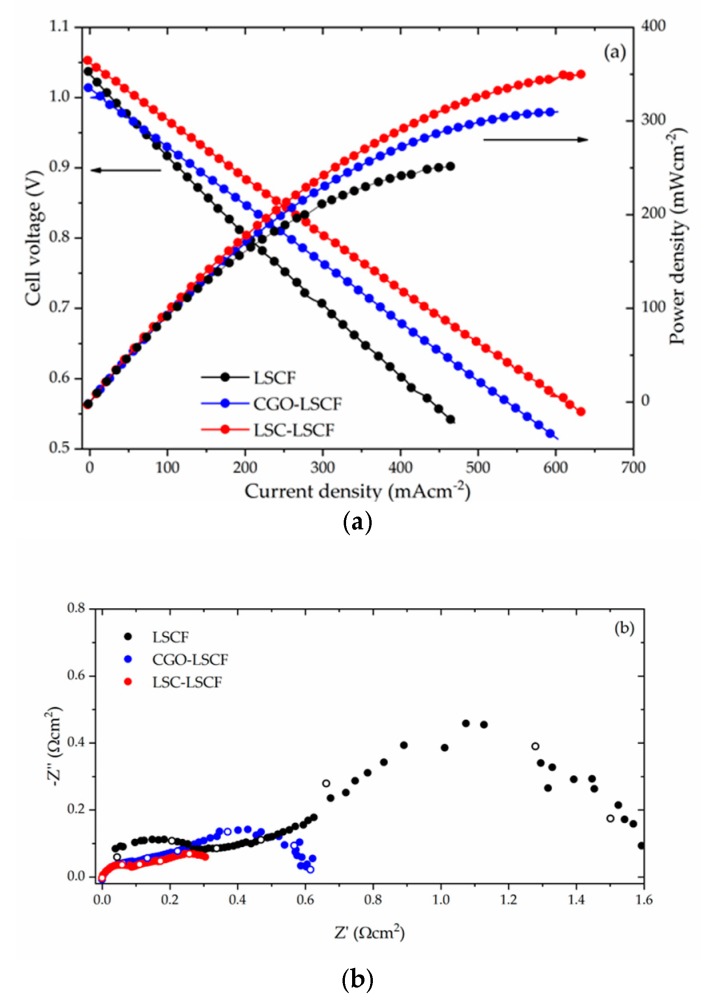

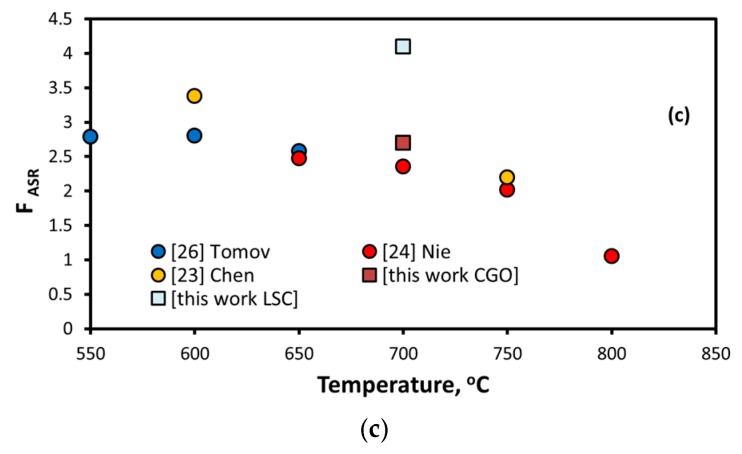
(**a**) I–V and I–P curves measured at 700 °C for the infiltrated and the reference cells; (**b**) relevant Nyquist plots at 700 °C (open points mark frequency decades between 10^6^ and 1 Hz); (**c**) A comparison of the *F_ASR_* values reported here with the ones published previously as a result of similar infiltration experiments.

Figure 7a shows the I–V and I–P curves measured at 700 °C for both infiltrated and non-infiltrated cells. The open cirquit voltage (OCV) values of the cells were similar and close to 1.04 V. The plots show that the infiltrated cells reached a higher maximum power density output, ~350 mW/cm^2^ for LCS infiltrated cell (*F_p_* = 1.40) and ~310 mW/cm^2^ for CGO infiltrated cell (*F_p_* = 1.24) in comparison to ~250 mW/cm^2^ for the reference cell. In addition, it can be noted that both infiltrated cells presented lower ohmic resistance—1.25 Ω (LSC infiltrated) and 1.05 Ω (CGO infiltrated)—with respect to the reference cell (2.14 Ω). This effect can be explained by the increased ionic conductivity in the infiltrated electrodes due to the additional contribution of CGO or LSC phases [46]. Direct observation of the polarization losses can be obtained from Figure 7b, where Nyquist plots measured at 700 °C are shown. To simplify the comparison, the ohmic part of impedance was subtracted. The Nyquist plots consisted of several suppressed overlapping arcs, showing the typical features of SOFC complete cell and representing the polarization losses taking place in both anodes and cathodes at different frequencies. The area specific resistances were estimated as ~1.6 Ω·cm^2^ for the reference cell, ~0.4 Ω·cm^2^ (*F_ASR_* = 4.00) for LSC-LSCF cell, and ~0.6 Ω·cm^2^ (*F_ASR_* = 2.7) for CGO-LSCF cell. An identification of multiple contributions appearing in the impedance spectra of the complete SOFC, shown in Figure 7b, is a non-trivial task. Systematic research using either simplified model configurations (e.g., half-cells) or change of parameters (e.g., variation of gases composition and temperature), often produced inconclusive results. The work of Leonide et al. [47] referred the origin of the low frequency losses to the gas–phase diffusion and the oxygen surface exchange kinetics/oxygen ion bulk diffusivity in frequency ranges of 0.1–1 Hz and 10–100 Hz, respectively. On the other hand, in a series of papers Mogensen’s group [48,49] assigned contributions at a higher frequency (~10 kHz) to the cathode side of the cell. The same approach was adopted in a very recent work by Shi et al which applied distribution of relaxation times (DRT)analysis [50]. Therefore, as far as the anodes were identically processed for all studied cells, it could be reasonably assumed that the variations in the polarization behavior can be ascribed to the differences in the infiltrated cathode electrodes.

The functionalization with CGO or LSC led to a significant reduction in the polarization resistance by introducing additional mechanisms promoting ORR. In the case of CGO nano decoration both m1 and m2 are possible (see Figure 1a), while the complete coverage with LSC suggests contribution from m3 (see Figure 1d). Such positive effect of CGO infiltration observed in MIEC LSCF cathode, having an already high ionic conductivity, is likely due to the fact that the ionic conductivity of CGO was observed to be higher than that of LSCF at intermediate SOFC temperatures [23,51]. Figure 7c provides a comparison between the *F_ASR_* values measured previously in similar infiltration experiments by several research groups with the ones obtained in this work. As could be seen a correspondence between several different infiltration experiments was established. The presented *F_ASR_* values are clearly grouped closely, with a tendency to increase at lower temperatures suggesting a repeatable effect of the infiltration procedure.

While both infiltrated nano decorations indicated an enhancement of the electrochemical activity, one should also take into account the possible effects of active surface area masking and porosity reduction. Quantification of these effects would require further experiments with small discrete changes of the inks loading levels.

## 8. Conclusions

A single step inkjet printing infiltration of CGO and LSC inks was implemented for the infiltration of LSCF cathodes. The cathode scaffolds’ surfaces were uniformly decorated with nano particles/nano coating, leading to an extension of the TPB and higher density of active sites promoting the ORR surface exchange reactions. The measured promotional factors of *Fp* = 1.24–1.40 and *F_ASR_* = 2.70–4.00 were comparable to the previously reported values in similar materials systems achieved with cumbersome manual procedures and at higher ink expenditure. Based on the analyzed data we speculate that the Sr segregation on the cathode scaffold surface is significantly reduced as a result of both infiltrations. Further direct evidence by appropriate quantitatave methods, like high resolution STEM-EDX and XPS, is needed in order to quantify the degree of Sr segregation suppression and clarify its mechanism. The IJP infiltration was shown to be a scalable and cost-effective alternative for an industrial scale-up of SOFC infiltration and surface modification procedures. The IJP infiltration brings a number of inherent advantages—high throughput due to the high frequency jetting, strict control of the jetted ink volumes, and uniformity with virtually no wastage of expensive ink precursors, as well as commercial availability of large scale printers.

## Figures and Tables

**Table 1:** Improvement factor (*F_ASR_*) comparison of doped ceria infiltrated La_0.6_Sr_0.4_Co_0.2_Fe_0.8_O_3−δ_ (LSCF) cathodes.

Source	Infiltrated Ink	Scaffold	T, °C	*F_ASR_*
Chen et al. [23]	Gd_0.2_Ce_0.8_O_2−x_	LSCF	600	3.66
			750	3.37
Nie et al. [24]	Sm_0.2_Ce_0.8_O_1.95_	LSCF	750	2.02
			800	1.05
Liu et al. [25]	La_0.4875_Ca_0.0125_Ce_0.5_O_2−δ_	LSCF	750	1.7
Tomov et al. [26]	Gd_0.1_Ce_0.8_O_2−x_	LSCF/CGO	650	2.56

**Table 2:** Basic characteristics of inkjet printing technologies.

	Piezoelectric	Electromagnetic
Drop volume	1–100 pL	10–50 nL+
Orifice size	10–60 µm	60–150 µm
Pressure regime	Vacuum (−50 mbar)	Positive (100–600 mbar)
Jetting distance	1 mm	4 mm
Maximum jetting rate	10–50 kHz	0.5–3 kHz
Multi-nozzles print head	~512	~16
Main advantage/Disadvantage	High resolution	Low cost robust technology
	High jetting rate	Ink compatibility
	High throughput	Disassembling/assembling/cleaning option
	High cost	Low resolution
Materials applications	Patterning	Coatings and infiltration
	Thin layers	Suspensions

**Table 3:** Infiltrate inks: compositions and jetting parameters. *

Ink	Viscosity, cP	Opening Time, µs	Drop Volume, nL	Drop Velocity, m·s^−1^
0.75 M CGO	4.6	250	38	2.3
0.75M LCO	4.2	250	32	2.4

* Pressure used for both inks: 350 mbar.
